# Biophysical Characterization of a Novel *SCN5A* Mutation Associated With an Atypical Phenotype of Atrial and Ventricular Arrhythmias and Sudden Death

**DOI:** 10.3389/fphys.2020.610436

**Published:** 2020-12-22

**Authors:** Mohammad-Reza Ghovanloo, Joseph Atallah, Carolina A. Escudero, Peter C. Ruben

**Affiliations:** ^1^Department of Biomedical Physiology and Kinesiology, Faculty of Science, Simon Fraser University, Burnaby, BC, Canada; ^2^Department of Pediatrics, Faculty of Medicine and Dentistry, University of Alberta, Edmonton, AB, Canada

**Keywords:** *SCN5A*, Nav1.5, channelopathy, atrial arrhymia, ventricular arrhythmia

## Abstract

**Background:**

Sudden cardiac death (SCD) is an unexpected death that occurs within an hour of the onset of symptoms. Hereditary primary electrical disorders account for up to 1/3 of all SCD cases in younger individuals and include conditions such as catecholaminergic polymorphic ventricular tachycardia (CPVT). These disorders are caused by mutations in the genes encoding cardiac ion channels, hence they are known as cardiac channelopathies. We identified a novel variant, T1857I, in the C-terminus of Nav1.5 (*SCN5A*) linked to a family with a CPVT-like phenotype characterized by atrial tachy-arrhythmias and polymorphic ventricular ectopy occurring at rest and with adrenergic stimulation, and a strong family history of SCD.

**Objective:**

Our goal was to functionally characterize the novel Nav1.5 variant and determine a possible link between channel gating and clinical phenotype.

**Methods:**

We first used electrocardiogram recordings to visualize the patient cardiac electrical properties. Then, we performed voltage-clamp of transiently transfected CHO cells. Lastly, we used the ventricular/atrial models to visualize gating defects on cardiac excitability.

**Results:**

Voltage-dependences of both activation and inactivation were right-shifted, the overlap between activation and inactivation predicted increased window currents, the recovery from fast inactivation was slowed, there was no significant difference in late currents, and there was no difference in use-dependent inactivation. The O’Hara-Rudy model suggests ventricular after depolarizations and atrial Grandi-based model suggests a slight prolongation of atrial action potential duration.

**Conclusion:**

We conclude that T1857I likely causes a net gain-of-function in Nav1.5 gating, which may in turn lead to ventricular after depolarization, predisposing carriers to tachy-arrhythmias.

## Introduction

Sudden cardiac death (SCD) is an unexpected death that occurs within an hour of the onset of symptoms (Myerburg and Junttila, 2012; Wellens et al., 2014). SCD is a devastating public health problem with major social implications and is responsible for thousands of deaths annually worldwide (Myerburg and Junttila, 2012). Hereditary primary electrical disorders account for up to a third of all SCD cases in younger individuals (Meyer et al., 2012) and include: long QT syndrome (LQTS), Brugada syndrome (BrS), short QT syndrome (SQT), and catecholaminergic polymorphic ventricular tachycardia (CPVT) (Priori and Chen, 2011; Pérez-Riera et al., 2018; Roston et al., 2018). These disorders are caused by mutations in the genes that encode cardiac ion channels, hence they are known as cardiac channelopathies.

CPVT is a malignant inheritable cardiac channelopathy that is triggered by stress or exertion and is characterized by atrial tachycardia, polymorphic ventricular arrhythmias and SCD. Most mutations in patients with CPVT have been reported in the gene that encodes the cardiac ryanodine receptor (*RyR2*). In addition to *RyR2*, few cases of CPVT have been caused by mutations in the genes that encode calsequestrin (*CASQ2*) and Kir2.1 (*KCNJ2*) (Priori and Chen, 2011; Pérez-Riera et al., 2018).

The sodium current passing through voltage-gated sodium channels (Nav) initiates action potentials in excitable cells. Nav channels are hetero-multimeric proteins composed of large ion conducting α-subunits and smaller auxiliary β-subunits (Isom et al., 1992; Catterall, 2012; Calhoun and Isom, 2014; Ghovanloo et al., 2016b, 2018; Ghovanloo and Ruben, 2020; Sait et al., 2020). The *SCN5A* gene encodes Nav1.5, which is the predominantly expressed Nav in the myocardium (Jiang et al., 2020). *SCN5A* mutations have been associated with LQTS, BrS, cardiac conduction defects, sick sinus syndrome, and dilated cardiomyopathy (Aiba, 2019). Additionally, Olesen et al. (2012) determined that a cohort of patients whose *SCN5A* mutations were previously associated with LQTS-(subtype 3) developed early onset lone atrial fibrillation. The biophysical characterization of these mutations suggested that there is considerable mechanistic overlap between LQTS-3 and early onset lone atrial fibrillation, which include alterations to peak currents, kinetics, and gating properties (Olesen et al., 2012).

This study focuses on a novel mutation, T1857I, in the C-terminus of Nav1.5 that is linked to a family with an inherited arrhythmogenic phenotype characterized by atrial tachy-arrhythmias and polymorphic ventricular arrhythmia occurring at rest and with adrenergic stimulation, and a strong family history of SCD, akin to but not typical of CPVT. We show full biophysical characterization of this mutation using whole-cell voltage-clamp.

## Materials and Methods

### Cell Culture

Chinese Hamster Ovary (CHOK1) cells were transiently co-transfected with cDNA encoding eGFP and the β1-subunit and either WT or variant Nav1.5 α-subunit. Transfection was done according to the PolyFect transfection protocol. After each set of transfections, a minimum of 8 h incubation was allowed before plating on sterile coverslips. The α:β1:eGFP ratio of transfection was 1:0.5:1.

### Electrophysiology

Whole-cell patch-clamp recordings were performed in an extracellular solution containing (in mM): 140 NaCl, 4 KCl, 2 CaCl_2_, 1 MgCl_2_, 10 HEPES (pH 7.4). Solutions were adjusted to pH 7.4 with CsOH. Pipettes were filled with intracellular solution, containing (in mM): 120 CsF, 20 CsCl, 10 NaCl, 10 HEPES. All recordings were made using an EPC-9 patch-clamp amplifier (HEKA Elektronik, Lambrecht, Germany) digitized at 20 kHz via an ITC-16 interface (Instrutech, Great Neck, NY, United States). Voltage-clamping and data acquisition were controlled using PatchMaster software (HEKA Elektronik, Lambrecht, Germany) running on an Apple iMac. Current was low-pass-filtered at 10 kHz. Leak subtraction was performed automatically by software using a P/4 procedure following the test pulse. Gigaohm seals were allowed to stabilize in the on-cell configuration for 1 min prior to establishing the whole-cell configuration. Series resistance was less than 5 MΩ for all recordings. Series resistance compensation up to 80% was used when necessary. All data were acquired at least 1 min after attaining the whole-cell configuration. Before each protocol, the membrane potential was hyperpolarized to −130 mV to ensure complete removal of both fast inactivation and slow inactivation. All experiments were conducted at 22°C. Glass pipette resistances were ∼1.5 MΩ.

### Activation Protocols

To determine the voltage-dependence of activation, we measured the peak current amplitude at test pulse potentials ranging from −100 to +80 mV in increments of +10 mV for 19 ms. Channel conductance (G) was calculated from peak I_Na_:

(1) $${\text{G}}_{\text{Na}}={\text{I}}_{\text{Na}}/\text{V}-{\text{E}}_{\text{Na}}$$

where G_Na_ is conductance, I_Na_ is peak sodium current in response to the command potential V, and E_Na_ is the Nernst equilibrium potential. Calculated values for conductance were fit with the Boltzmann equation:

(2) $$\text{G}/{\text{G}}_{\max}=1/\left(1+\exp\left[-{\text{ze}}_{0}\,\left[{\text{V}}_{\text{m}}-{\text{V}}_{1/2}\right]/\text{kT}\right]\right)$$

where G/G_max_ is normalized conductance amplitude, V_m_ is the command potential, z is the apparent valence, e_0_ is the elementary charge, V_1__/__2_ is the midpoint voltage, k is the Boltzmann constant, and T is temperature in K.

### Steady-State Fast Inactivation Protocols

The voltage-dependence of fast inactivation was measured by preconditioning the channels to a hyperpolarizing potential of −130 mV and then eliciting pre-pulse potentials that ranged from −170 to +10 mV in increments of 10 mV for 200 ms, followed by a 10 ms test pulse during which the voltage was stepped to 0 mV. Normalized current amplitudes from the test pulse were fit as a function of voltage using the Boltzmann equation:

(3) $$\text{I}/{\text{I}}_{\max}=1/(1+\exp\left(-{\text{ze}}_{0}\left({\text{V}}_{\text{M}}-{\text{V}}_{1/2}\right)/\text{kT}\right)$$

where *I*_max_ is the maximum test pulse current amplitude.

### Recovery From Fast Inactivation Protocols

Channels were fast-inactivated during a 20 or 200 ms depolarizing step to 0 mV, and recovery was measured during a 19 ms test pulse to 0 mV following a −90 mV recovery pulse for durations between 0 and 1.024 s. Time constants of fast inactivation recovery showed two components and were fit using a double exponential equation:

(4) $$\text{I}=\mathrm{Iss}+\alpha_{1}\,\exp\left(-\text{t}/\tau \,1\right)+\alpha_{2}\,\exp\left(-\text{t}/\tau \,2\right)$$

where *I* is current amplitude, *I*_ss_ is the plateau amplitude, α_1_ and α_2_ are the amplitudes at time 0 for time constants τ_1_ and τ_2_, and *t* is time.

### Use-Dependent Inactivation −3 Hz

Channels accumulated into a use-dependent inactivated state during either a series of depolarizing pulses to 0 mV. Normalized current amplitude as a function of time was fitted with a double exponential.

### Late Current Protocol

Late current was measured between 145 and 150 ms during a 200 ms depolarizing pulse to 0 mV from a holding potential of −130 mV. Pulses were averaged to increase signal-to-noise ratio.

### Window Current Measurements

Window current areas were analyzed by converting activation and inactivation curves to precents (Wang et al., 1996) and calculating the area under both curves by integration using Microsoft Excel; the position of area peak was estimated in Igor Pro.

### Action Potential Modeling

Ventricular action potentials were simulated using a modified version of the O’Hara–Rudy model programmed in Matlab (O’Hara et al., 2011; Fouda et al., 2020). The code that was used to produce the model is available online from Washington University in St. Louis^1^. The modified gating INa parameters were in accordance with the most relevant biophysical data obtained from whole-cell patch-clamp experiments in this study for various conditions, including voltage-dependences of activation, inactivation, and peak sodium conductance. Atrial action potentials were simulated using a modified version of the Grandi model coded in Matlab (Grandi et al., 2011; Morotti et al., 2016). The original code was acquired from UC Davis^2^. Original equations for O’Hara-Rudy:

(5) mss= 1.0/(1.0+exp⁢((-(v+ 39.57))/9.871))

(6) hss= 1.0/(1.0+exp⁢((v+ 82.9)/6.086))

and Grandi:

(7) $$\text{mss}=1.0/\left({\left(1.0+\exp\left(-\left(56.86+\text{v}\,\left(39\right)\right)/9.03\right)\right)}^{2}\right)$$

(8) $$\text{hss}=1.0/\left({\left(1.0+\exp\left(\left(\text{v}\,\left(39\right)+71.55\right)/7.43\right)\right)}^{2}\right)$$

The modifications to the original parameters where based the magnitude differences between WT and T1557I shown in Supplementary Tables S1–S6.

### Analysis

Analysis and graphing were done using FitMaster software (HEKA Elektronik) and Igor Pro (Wavemetrics, Lake Oswego, OR, United States). All data acquisition and analysis programs were run on an Apple iMac (Apple Computer). Statistical analysis was performed in JMP version 13.

### Statistics

Continuous variables are presented as means ± standard error in mean and were normally distributed. *T*-test was used to compare the mean responses (activation, current density, steady-state fast inactivation, late currents, use-dependent inactivation, and fast inactivation recovery) between channel variants (two levels: WT and T1857I). A level of significance α = 0.05 was used in all overall tests, and effects with *p* < 0.05 were considered statistically significant.

## Results

### Phenotypic Characterization

**FIGURE 1:**
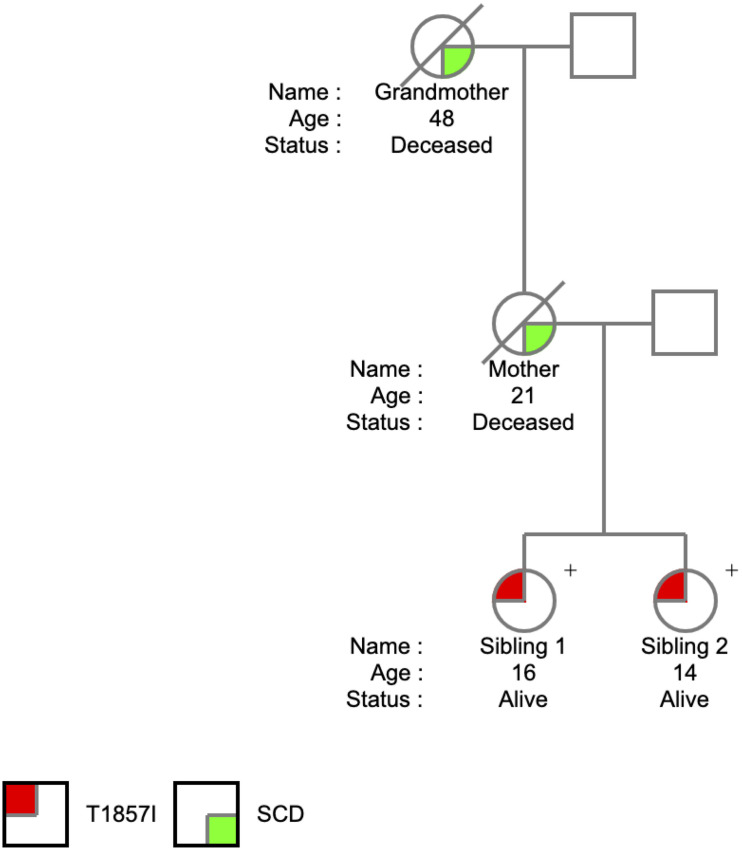
Pedigree of the T1857I Variant in Nav1.5.

The T1857I mutation was identified in two sisters at the ages of 14 and 16 years. The *SCN5A* variant was identified on a 165 comprehensive cardiac next generation sequencing (NGS) gene panel. The panel included known arrhythmia and cardiomyopathy genes. There was no history of arrhythmic syncope or seizure. The family history (Figure 1) was relevant for SCD in the mother at the age of 21 years and in the maternal grandmother at the age of 48 years, both occurring at rest. The remaining maternal family history is relevant for multiple individuals with early onset atrial fibrillation and SCD at a young age. Investigations included serial ECGs, signal average ECGs, high-lead ECGs, exercise stress tests, 24 h Holter monitoring, echocardiograms, and cardiac magnetic resonance imaging. These investigations demonstrated a pattern of progressively increasing burden of atrial and ventricular ectopy and non-sustained tachycardia of post-pubertal onset. The arrhythmias worsened with exercise but were also ambient at baseline. Criteria were not met for any cardiac channelopathy, specifically a normal QTc at baseline and in recovery on exercise stress test, no evidence of Brugada like changes, no pathologic early repolarization, as well as no evidence of cardiomyopathy. The clinical characteristics were suggestive of an atypical CPVT-like phenotype. A broad arrhythmia genetic screening panel was performed, identifying the novel T1857I mutation in both siblings.

**FIGURE 2:**
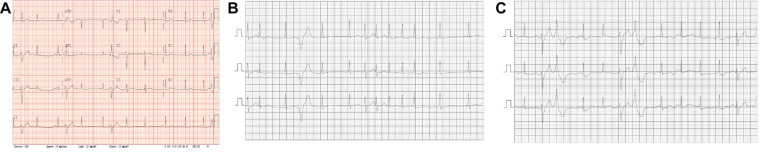
ECGs/Relevant Clinical Data. **(A)** Standard 12-lead ECG obtained from the patient with the novel *SCN5A* mutation. Sinus rhythm with frequent polymorphic premature wide complex beats, low T-wave amplitude and inverted T-waves in the right precordial leads (V1 and V2), normal axis and QRS duration and QTc = 396. **(B,C)** Segments from 24 h Holter monitoring. **(B)** Shows a non-sustained irregular narrow complex tachycardia suspected to be atrial tachycardia. **(C)** Shows bi-directional PVCs.

During 5 years of follow-up, both siblings developed a very similar phenotype with frequent catecholamine sensitive atrial and polymorphic ventricular ectopy with non-sustained atrial tachycardia up to 200 bpm along with bidirectional PVCs and polymorphic triplets. The arrhythmias are only partially responsive to Nadolol and were thought to be minimally responsive to Flecainide. A sample ECG is shown in Figure 2A showing sinus rhythm with frequent polymorphic premature wide complex beats, depolarization abnormality with low T-wave amplitude and inverted T-waves in the right precordial leads (V1 and V2), and otherwise normal intervals. Twenty-four-hour Holter monitoring revealed patterns consistent with CPVT-like pathology. Figure 2B shows a non-sustained irregular narrow complex tachycardia suspected to be atrial tachycardia, and Figure 2C shows bidirectional PVCs, which usually raise the suspicion for CPVT.

### T1857I Depolarizes Activation, Increases Apparent Valence

**FIGURE 3:**
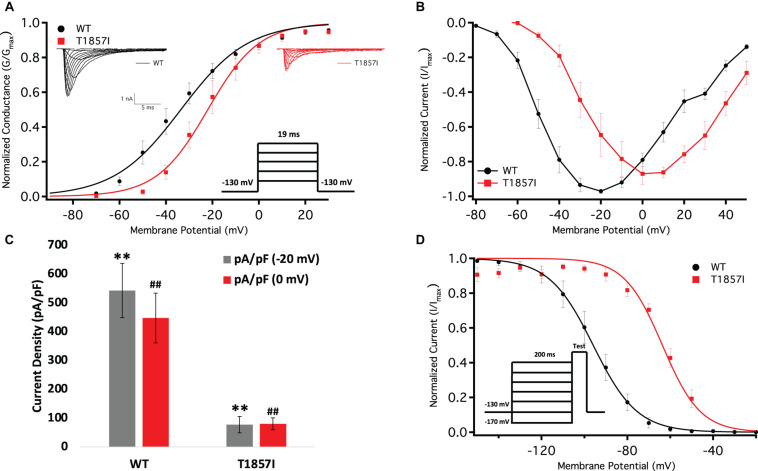
Comparison of the Voltage-Dependence of Activation/Fast Inactivation Between WT and T1857I. **(A)** Voltage-dependence of activation as normalized conductance plotted against membrane potential. Sample macroscopic sodium currents elicited by depolarizations between −100 and +80 mV (reversal potential: WT = 49.7 ± 4 mV, TI = 59.5 ± 2 mV). **(B)** Normalized current and voltage relationship. Each recording was normalized relative to its own maximal peak value. The normalized current and voltage relationships shown are averaged for each condition. **(C)** Average current (*Y*-axis) density of WT and T1857I. **(D)** Voltage-dependence of steady-state fast inactivation as normalized current plotted against membrane potential. ** and ^##^ represent statistical significance.

We conducted whole-cell patch-clamp experiments to investigate whether the T1857I variant in the C-terminus alters the biophysical properties of Nav1.5. We examined the variant effect on channel activation in WT and T1857I by measuring peak channel conductance at membrane potentials between −100 and +80 mV (Figures 3A,B). We found that the mutation causes a significant shift on the midpoint (V_1__/__2_) of the conductance curve (*p* = 0.0069) in the depolarized direction. T1857 also causes a significant increase in the apparent valence (z) of activation (*p* = 0.0317) (Supplementary Table S1). This suggests that this C-terminal mutation has a destabilizing effect on the voltage-dependence of activation and also affects magnitude of charge movement during activation.

### T1857I Decreases Peak Current Density

We measured current density from the ratio of peak current amplitude to the cell membrane capacitance (pA/pF) (Figure 3C). As variant activation curve was right-shifted compared to WT, we measured the peak current densities at −20 mV (where WT elicits maximal current) and 0 mV [where T1857I elicits maximal current (Figure 3B)]. Our results suggested that the T1857I variant channels had significantly smaller current densities than WT at both potentials (*p* < 0.0001) (Figure 3C and Supplementary Table S2).

### T1857I Depolarizes Steady-State Fast Inactivation (SSFI)

We measured the voltage-dependence of SSFI using a standard 200 ms pre-pulse voltage protocol. Normalized current amplitudes were plotted as a function of pre-pulse voltage (Figure 3D and Supplementary Table S3). Our results indicate T1857I significantly destabilizes the V_1__/__2_ (*p* < 0.0001), but not the z (*p* > 0.05) of the voltage-dependence of SSFI. This indicates that at any given potential the variant channels are less likely to fast inactivate, suggesting an increased excitability.

### T1857I Slows Recovery From Inactivation

**FIGURE 4:**
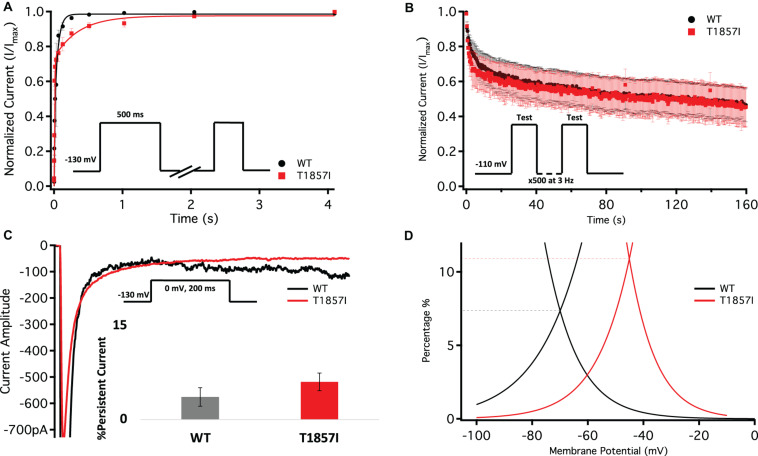
Comparison of Fast Inactivation Recovery, Use-Dependence, Persistent Currents, and Window Currents. **(A)** Recovery from fast inactivation. **(B)** Use-dependent inactivation at 3Hz. Show normalized current decay plotted as a function of time. **(C)** Representative current traces of late currents in WT and T1857I. Average late sodium current as a percentage of peak sodium current. **(D)** Window currents obtained from the overlap of the GV and SSFI curves shown in Figures 3A,D. The curves shown are the Boltzmann fits of the data. The dashed lines show the approximate window peak, on a *Y*-axis that is converted to percentage as described in Wang et al. (1996). The peak for WT is reached at −70 mV and 7.4%, and for T1857I at −45 mV and 10.9%. The window area for T1857I is about 62% bigger than WT.

One benchmark of Nav is the rate at which they recover from inactivation. To measure recovery, we held Nav1.5 at −130 mV to ensure channels were fully available, then pulsed the channels to 0 mV for 500 ms and allowed different time intervals at −130 mV to measure recovery as a function of time (Figure 4A and Supplementary Table S4). Our results indicate that T1857I variants have a relatively slowed rate of recovery from fast inactivation (*p* < 0.05).

### T1857I Does Not Alter Use-Dependent Inactivation

To understand the effects of elevated heart rate on channel function, we elicited use-dependent inactivation at 3 Hz. Normalized currents from 3 Hz use-dependent inactivation protocols are plotted against time in Figure 4B for both the channel variants. We found that there are no significant differences between WT and T1857I in use-dependent inactivation rates (*p* > 0.05) (Supplementary Table S5).

### T1857I Does Not Alter Late Currents, Increases Window Currents

Total sodium current can be divided into two components: peak (INaP) and late (INaL) currents. Whereas INaP refers to the maximum amount of sodium ions going through the channels during the open state, the smaller INaL is a manifestation of destabilized fast inactivation and has been shown to underlie LQT syndromes (Wang et al., 1995; Jarecki et al., 2010; Ghovanloo et al., 2016a; Ghovanloo and Ruben, 2020). We show representative current traces for both channels across all conditions (Figure 4C and Supplementary Table S6). We measured the percentage of INaL by dividing the maximum INaL between 145 and 150 ms by INaP and found that there is no significant difference in the percentage of late currents between WT and T1857I. This is consistent with the normal QTc observed in the patient.

Changes in the voltage-dependence of both steady-state activation and fast inactivation may lead to differences in the window current. Consistent with the large right-shift of the SSFI curve, we found that T1857I has an increased sodium window current compared with WT-Nav1.5. We also found that the window current period was right-shifted in T1857I compared to WT (Figure 4D).

### T1857I Causes After Depolarizations in the O’Hara–Rudy Model of Ventricular Cardiac Excitability

**FIGURE 5:**
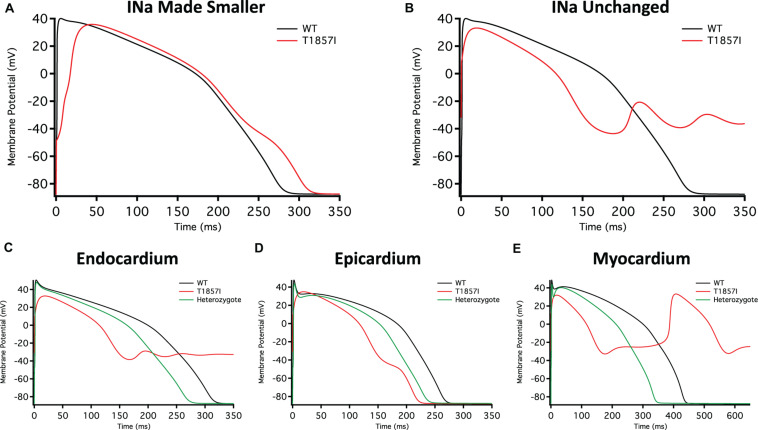
O’Hara-Rudy-Based Ventricular Action Potential Model. Comparison of the action potential between WT and T1857I. The changes to conductances were based on experimental data obtained from patch-clamp results. **(A)** Changed gNa, **(B)** Unchanged gNa. **(C–E)** Endocardium/epicardium/myocardium simulations with WT, T1857I, and Heterozygote (unchanged gNa).

We used the O’Hara–Rudy model to simulate cardiac action potentials (AP) (pacing cycle length 1,000 ms) and measure the effect of the T1857I mutation (O’Hara et al., 2011). The control results from the patch-clamp experiments were adjusted to the original model parameters and the subsequent magnitude shifts in the simulations of the mutation were performed relative to the original model parameters (Figure 5).

The smaller current densities in T1857I (Figure 3C) could have two possible underlying causes: (1) the variant biophysically alters and reduces the sodium conductance, (2) the variant construct has a smaller expression/trafficking, hence the smaller sodium conductance in patch-clamp experiments is an artificial effect. Therefore, we performed two sets of simulations for T1857I. In Figure 5A, we show an AP simulation where we reduced the sodium conductance by sixfold, which is consistent with the current density data shown in Figure 3C. The results from this condition show reduction in the upstroke velocity and peak amplitude of phase 0, and a slight after depolarization hump that starts at ∼250 ms. In Figure 5B, we left the sodium conductance unchanged in T1857I. In this simulation, a more pronounced after depolarization was observed starting at ∼225 ms. Overall, both sets of simulations suggest that T1857I causes after depolarizations, which could lead to cardiac arrhythmias.

To examine possible variant effects on different ventricular cell types, we compared simulated APs between endocardium, epicardium, and myocardium. After depolarizations are observed in all three cell types (Figures 5C–E). Also, as our patients are heterozygotes (maternal inheritance of the variant allele), we performed all simulations with a heterozygous assumption (i.e., 50% of the channels have the variant phenotype and 50% are WT). The AP morphology of the heterozygous simulations appear less exaggerated than the pure T1857I conditions, and therefore may be a better representation of ventricular excitability in our patients, congruent with the normal QRS duration on baseline ECG in both patients.

### T1857I Causes Slight GOF in Atrial Grandi Model of Cardiac Excitability

**FIGURE 6:**
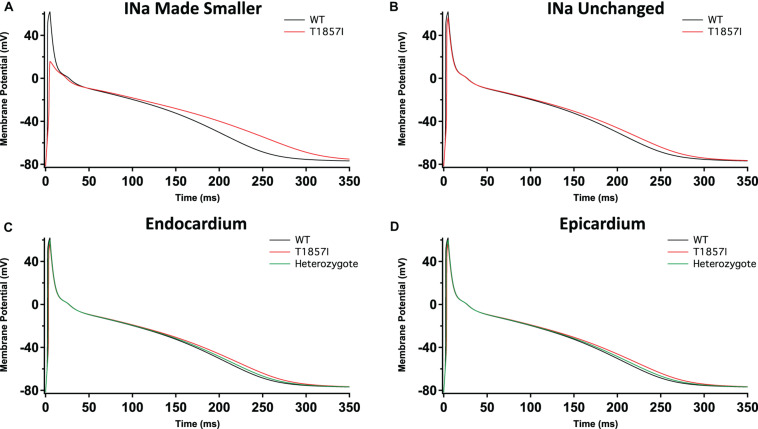
Grandi-Based Atrial Action Potential Model. Comparison of the action potential between WT and T1857I. The changes to conductances were based on experimental data obtained from patch-clamp results. **(A)** Changed gNa, **(B)** Unchanged gNa. **(C,D)** Endocardium/epicardium simulations with WT, T1857I, and heterozygote (unchanged gNa).

To investigate possible T1857I effects on atrial excitability, we ran atrial AP simulations using the Grandi model (Grandi et al., 2011; Figures 6A–D). Similar to the ventricular APs, reducing the sodium conductance lowered the AP peak and slightly prolonged the APD (Figure 6A). However, in the simulations in which the sodium conductance remained unchanged, the variant effects were minimal (Figure 6B). Furthermore, there were virtually no differences in the variant effect between atrial epicardium and endocardium simulations (Figures 6C,D), and these effects became even more subtle in heterozygote conditions. Overall, based on the Grandi model, the effect of T1857I on atrial excitability is predicted to be minimal.

## Discussion

### Possible Underlying Mechanisms and Implications

We identified a novel variant, T1857I, explored its effects on the biophysical properties of Nav1.5, and used the results to simulate cardiac action potentials. Our results suggest that the voltage-dependence of both activation and inactivation are right-shifted. The right-shift of activation suggests a loss-of-function, in contrast the larger right-shift of inactivation suggests a gain-of-function in this channel. We also found that the recovery from fast inactivation was slowed. To determine the effects of gating shifts in T1857I, we first introduced the relevant experimental data into the ventricular O’Hara-Rudy model, which suggests after depolarizations may result. We then performed atrial AP modeling using the Grandi model, which suggested minimal variant effects. Thus, we conclude that T1857I likely causes a net gain-of-function in the Nav1.5 gating, with an increased window current and resultant ventricular tissue after depolarization, which could lead to arrhythmias. These biophysical properties of after depolarization, unaltered late Na^+^ current and unchanged AP duration are, respectively, consistent with the clinical phenotype of the probands characterized by frequent and polymorphic ventricular ectopy and tachy-arrhythmia, normal QTc and QRS duration.

The inward sodium current through Nav1.5 is the principal determinant of V_max_ inside the myocardium. The functional differences in human atrial and ventricular sodium current biophysical properties were suggested to be minimal (Sakakibara et al., 1992, 1993; Jia et al., 1993; Furukawa et al., 1995). Although Nav1.5 is the predominant Nav channel in the heart, the transcript for the sodium channel auxiliary β1-subunit is more highly expressed in the atrium (Gaborit et al., 2007). Recent structural studies determined the structural basis for a reduced modulation of Nav1.5 by β1-subunits. They suggested that the sodium current amplitude differences between atrial and ventricular cells are unlikely to be significantly affected by β1-subunits expression levels (Ghovanloo and Ruben, 2020; Jiang et al., 2020). Additionally, reports in canine atrial cells suggest that the atria have a larger sodium current density and a hyperpolarized inactivation compared to ventricles (Burashnikov et al., 2007). Also, due to the depolarized resting membrane potential, there is less sodium channel availability in the atria compared to the ventricle, and hence, the resulting sodium current underlying the atrial action potential is smaller in atrial cells (Gui-Rong and Nattel, 1997). In contrast to the sodium current, several other ionic currents display more pronounced differences. These differences underly the difference in action potential morphology between these cell types (Pandit, 2018). These differences may also be the reason for the less pronounced T1857I experimentally simulated effects in atria compared to ventricles (Figures 5, 6). Our modeling did not account for recovery from inactivation, which is a limitation of our simulations.

The sodium/calcium exchanger (NCX) plays a vital role in maintaining cellular calcium levels. NCX takes advantage of higher extracellular sodium concentrations to pump calcium out of the cytosol. However, the NCX also functions in reverse mode (Satoh et al., 2000; Priori and Chen, 2011). The human atrial action potential is also modulated by intracellular calcium concentrations, which can directly affect calcium channel inactivation (Nattel, 1997) that modulates the timing and size of NCX current (Bénardeau et al., 1996; Li and Nattel, 1996). Our results suggest that the most notable biophysical property of T1857I is its ∼30 mV right-shifted SSFI. This large shift increases the overlap (window current) between the SSFI and activation curves in T1857I. The elevated window current may explain the after depolarizations observed in our AP simulations. Given this SSFI curve, at any given membrane potential, more sodium influx into the cytosol is expected. In turn, this may induce reverse mode NCX activity leading to Ca^2+^ entry and give rise to after depolarizations in both atrial and ventricular myocytes. This hypothesis merits further investigations through measurement of NCX activity in cardiac myocytes.

The phenotypic expression of T1857I is similar to that of R222Q, which underlies multifocal ectopic Purkinje-related premature contractions (MEPPC) (Laurent et al., 2012). Both disorders include atrial arrhythmias, premature ventricular contractions, and may result in SCD. However, the biophysical underpinnings of the two syndromes are quite different. Although both R222Q and T1857I cause a depolarizing shift in the voltage-dependence of activation, our results with T1857I also show a depolarizing shift in SSFI, whereas R222Q causes a small hyperpolarizing shift in inactivation. Furthermore, the *in silico* action potentials have different morphologies arising from the two mutations. A more recent study reported another MEPPC-causing mutation, G213D, that similar to T1857I right-shifted inactivation; however, unlike T1857I, the G213D activation curve was left-shifted (Calloe et al., 2018). These differences underscore the novelty of our clinical and biophysical observations related to T1857I.

### Rationale for Using CHO Cells for T1857I Characterization

In this study, our experiments were performed using CHOK1 cells, which are a heterologous expression system. CHO cells have small background currents and are excellent for studying channels using transient transfections. Navs are typically modulated by several proteins and accessory subunits which could be missing in heterologous systems. However, despite these limitations, heterologous systems work like blank canvases that are the gold standard for purely biophysical studies such as those presented here. Although induced pluripotent stem cells (iPSC) are generally considered more physiological than CHO cells, they do not fully recapitulate intact human cells (Knollmann, 2013; Goversen et al., 2018). For instance, iPSC-derived cells are often immature and lack various critical structures (Knollmann, 2013; Goversen et al., 2018). Furthermore, these cells are inadequate for studying the biophysical properties of any given channels and may therefore reduce reproducibility. Furthermore, studying the properties of a single channel type (in this case to isolate Nav1.5) requires using elaborate pharmacological cocktails which may introduce confounding effects. To that end, to reduce the confounding effects of the complex genetic background and need for extensive channel blockers, we performed our studies in the simple CHO cells, which enabled a deep understanding of the electrophysiology of T1857I, and we applied that info to well-established cardiac models to visualize the mutant effects on gating.

## Conclusion

In conclusion, we report a novel *SCN5A* mutation encoding T1857I where afflicted probands present with clinically characterizable incidence of atrial and ventricular arrhythmias and multiple familial sudden deaths. The biophysical characterization of this variant indicates a mixture of gain- and loss-of-function effects. Computer simulations using the experimental data suggest after depolarizations as the net result. These mechanistic findings are consistent with the clinically observed cardiac arrhythmias in the affected probands.

## Data Availability Statement

The raw data supporting the conclusions of this article will be made available by the authors, without undue reservation.

## Ethics Statement

The studies involving human participants were reviewed and approved by Alberta Health Services Research Ethics Board. Written informed consent to participate in this study was provided by the participants’ legal guardian/next of kin.

## Author Contributions

M-RG performed all functional experiments, action potential modeling, data analysis, figure making, and wrote manuscript. PCR conceived the experiments and revised the manuscript critically. JA and CAE performed all clinical work. All co-authors edited the manuscript.

## Conflict of Interest

The authors declare that the research was conducted in the absence of any commercial or financial relationships that could be construed as a potential conflict of interest.

## Footnotes

1 https://wustl.app.box.com/s/o404cypdy04p49zgr4zhxoma5a75cesb/file/580713267432

2 https://somapp.ucdmc.ucdavis.edu/Pharmacology/bers/
