# Supplementary material for: Biophysical Characterization of a Novel SCN5A Mutation Associated With an Atypical Phenotype of Atrial and Ventricular Arrhythmias and Sudden Death
Source: Front Physiol. 2020 Dec 22;11:610436. doi: 10.3389/fphys.2020.610436 (PMC7783455; doi:10.3389/fphys.2020.610436)
Supplement: Supplementary file 1 [file Table_1.docx]

**Supporting Information**

**Table S1- Conductance (n = 6-9)**

| **Channel Type** | **Mean V_1/2_ ± SE (mV)** | **Mean z ± SE (slope)** |
| --- | --- | --- |
| WT | -35.8 ± 3.8 | 2.3 ± 0.3 |
| T1857I | -20.2 ± 3.1* | 3.1 ± 0.2* |

^*^ Statistical significance (p-value provided in text)
