# Supplementary material for: Biophysical Characterization of a Novel SCN5A Mutation Associated With an Atypical Phenotype of Atrial and Ventricular Arrhythmias and Sudden Death
Source: Front Physiol. 2020 Dec 22;11:610436. doi: 10.3389/fphys.2020.610436 (PMC7783455; doi:10.3389/fphys.2020.610436)
Supplement: Supplementary file 2 [file Table_2.docx]

**Table S2- Current Density (n = 7-11)**

| **Channel Type** | **Mean density (-20 mV) ± SE (pA/pF)** | **Mean density (0 mV) ± SE (pA/pF)** |
| --- | --- | --- |
| WT | 543 ± 64 | 447 ± 57 |
| T1857I | 77 ± 51* | 80 ± 45* |

^*^ Statistical significance (p-value provided in text)
