# Supplementary material for: Biophysical Characterization of a Novel SCN5A Mutation Associated With an Atypical Phenotype of Atrial and Ventricular Arrhythmias and Sudden Death
Source: Front Physiol. 2020 Dec 22;11:610436. doi: 10.3389/fphys.2020.610436 (PMC7783455; doi:10.3389/fphys.2020.610436)
Supplement: Supplementary file 4 [file Table_4.docx]

**Table S4- Fast Inactivation Recovery (n = 3-6)**

| **Channel Type** | **Mean** 𝞃_1_ **± SE (s)** | **Mean** 𝞃_2_ **± SE (s)** |
| --- | --- | --- |
| WT | 0.0057 ± 0.004 | 0.35 ± 0.18 |
| T1857I | 0.0063 ± 0.001* | 0.050 ± 0.021* |

^*^ Statistical significance (p-value provided in text)
