# Supplementary material for: Biophysical Characterization of a Novel SCN5A Mutation Associated With an Atypical Phenotype of Atrial and Ventricular Arrhythmias and Sudden Death
Source: Front Physiol. 2020 Dec 22;11:610436. doi: 10.3389/fphys.2020.610436 (PMC7783455; doi:10.3389/fphys.2020.610436)
Supplement: Supplementary file 5 [file Table_5.docx]

**Table S5 - 3Hz (n = 7-8)**

| **Channel Type** | **Mean** 𝞃 **± SE (s)** |
| --- | --- |
| WT | 12.3 ± 3.0 |
| T1857I | 9.0 ± 2.8 |
