# Supplementary material for: Biophysical Characterization of a Novel SCN5A Mutation Associated With an Atypical Phenotype of Atrial and Ventricular Arrhythmias and Sudden Death
Source: Front Physiol. 2020 Dec 22;11:610436. doi: 10.3389/fphys.2020.610436 (PMC7783455; doi:10.3389/fphys.2020.610436)
Supplement: Supplementary file 6 [file Table_6.docx]

**Table S6 – INaL (n = 3-7)**

| **Channel Type** | **Mean % ± SE (mV)** |
| --- | --- |
| WT | 3.6 ± 1.5 |
| T1857I | 6.0 ± 1.0 |
